# Tubercular Joint Disease in Children—A General Consideration*A clinical lecture delivered at Randall’s Island Hospital. Reported by Geo. H. Semkie.

**Published:** 1895-01

**Authors:** Samuel E. Milliken

**Affiliations:** New York; Surgeon-in-Chief of the New York Infirmary for Crippled Children; Surgeon to Randall’s Island Hospital, &c., &c.


### Southern Medical Record.

A MONTHLY JOURNAL OF MEDICINE AND SURGERY.

VOL. XXV. ATLANTA, GA., JANUARY, 1895. No. 1.

### Original Articles.

### TUBERCULAR JOINT DISEASE IN CHILDREN—A GENERAL CONSIDERATION.*

BY SAMUEL E. MILLIKEN, M. D.,

Surgeon-in-Chief of the New York Infirmary for Crippled Children; Surgeon to Randall's Island Hospital, &c., &c.

Before entering into the consideration of the differential diagnosis and treatment of individual joints, it might be well to speak of these tubercular lesions in a general way. First, let us understand the various stages of this very important branch of surgery in childhood, and in what respect it differs from a disease produced by the same bacillus later in life.

First: A primary tubercular synovitis is much less frequent in children than in adults, for this disease usually affects the epiphyseal end of the bone first and, if recognized in time, abscess and the disagreeable sinuses may be avoided and the course of the disease can often be materially shortened if prompt measures be instituted.

Second: ATROPHY.—Before any signs of abscess and when little or no deformity exists, there will be found an appreciable atrophy of the muscles to have taken place; this alone should lead us to look upon the case as suspicious.

Third: LIMP.—The limp, surprising to say, is variable and this fact alone, no doubt, leads many to pass the case with

*A clinical lecture delivered at Randall's Island Hospital. Reported by Geo. H. Semkie.

only a superficial examination, but to administer some anti-rheumatic, while the family are very willing to consider the child only suffering from the usual "growing pains."

Fourth: RESTLESSNESS,—Restlessness (and the "night cries" where the hip joint is involved) should assist materially when of frequent occurrence, but must be combined with a most careful physical examination of the joint or regions.

Fifth: MUSCULAR SPASM.—Spasm of the surrounding muscles, although very slight during the incipiency, should be carefully noted, but when there is a recent history of traumatism, we should not be too hasty in making a diagnosis of bone disease. While there is no doubt but that injury is often a contributing cause, clinical data are quite sufficient to convince us that the bacillus tuberculosis is always to be found.

Sixth: ABSCESS.—Abscess of tubercular origin may not be recognized until the bone lesion has existed months, or even more than a year; however, its presence should be detected early. It should not be forgotten that periarticular abscesses not infrequently follow many of the exanthemata and when so, prompt relief should be given lest the joint become involved secondarily. The recognition of a central ostitis of the head of the tibia may also prevent the subsequent involvement of the joint. I recall such a case which was operated upon four and a half years ago, where there existed a cavity the size of a small egg filled with tubercular material, and yet the joint has remained free from disease.

TREATMENT.—The most important factor to be met in the treatment of tubercular joint lesions is that of *protection*. Each orthopædic surgeon seems to feel that a different apparatus meets this demand best. The Englishman seems to think that the immobilization splint of Thomas does the work best, while Americans rather tend towards the idea of extension. A few surgeons have endeavored to make us believe that both immobilization and extension are required to obtain the best results.

Common sense should teach us that concussion or anything that produces the least possible injury to the involved bone must be avoided until repair shall have taken place.

To hasten this process absolute rest of the joint should be maintained, and this can best be accomplished by placing the child in bed while a careful study of the case is made. When the diagnosis is clear, it is my practice to first immobilize the joint with plaster of paris, and where any great amount of muscular spasm is found, a moderate extension is applied.

The great mistake so often made by the specialist is the concentration of all his forces on the particular part of the human economy in which he is most interested, while the general health is allowed to take care of itself. No class of cases require more detail in their general treatment than do these tubercular bone lesions. In all cases where suppurating sinuses have existed for any length of time, we should not fail to investigate the condition of the kidneys, lest amyloid changes occur. The digestive and assimilative powers are invariabiy weak, for which we should administer some form of bitter tonic and at the same time give reconstructives to compensate for the waste which has already taken place. While cod liver oil and hypophosphites are often prescribed, I have recently obtained most flattering results in suppurating joint cases in children, even after they were bed-ridden, with equal parts of pure cod liver oil and maltine with coca wine. The assimilative powers are very much strengthened, and with some of the worst cases even, the improvement was noticed within a very short time. Maltine with coca wine is an excellent vehicle for cod liver oil and its food and digestive values, combined with the peculiar tonic properties of the coca, makes it a very useful aid in the treatment of this class of cases.

---

MODERN SURGICAL TECHNIQUE.—Dr. Henry O. Marcy, of Boston, emphasizes the importance of a most careful bacteriological training on the part of him who would become proficient in suigical practice. In the preparation of the operating-room, Dr. Marcy pointed out the ease and safety with which an ordinary living-room, by preference the kitchen, is made comparatively sterile, when from necessity, the surgeon is called upon to act promptly and suddenly. In abdominal wounds, where irrigation is not advised, he substitutes for it a slowly flowing stream of oxygen gas from a compressed cylinder. This sterile gas is heavier than atmospheric air; which it displaces, and as a consequence, renders the wound less likely to infection from the products of respiration and atmospheric contaminations.—From report of Mississippi Valley Medical Association in *Medical Record.*

